# Breathless Balance: Navigating Pembrolizumab-Induced Pneumonitis and Congestive Heart Failure (CHF) Exacerbation

**DOI:** 10.7759/cureus.87150

**Published:** 2025-07-02

**Authors:** Philip Hall, Catherine Miller, Arsalan Ahmad, Karim Akl, Mikhail Litinski

**Affiliations:** 1 Internal Medicine, Bayonne Medical Center, Bayonne, USA; 2 Pulmonary and Critical Care Medicine, Jersey City Medical Center, Jersey City, USA; 3 Pulmonary and Critical Care Medicine, Bayonne Medical Center, Bayonne, USA

**Keywords:** cardiopulmonary toxicity, congestive heart failure, immune checkpoint inhibitor, pembrolizumab, pneumonitis

## Abstract

Immune checkpoint inhibitors like pembrolizumab have transformed oncology but carry the risk of immune-related adverse events (irAE), including pneumonitis. We report a case of a 66-year-old male with multiple cardiac comorbidities who presented with acute hypoxic respiratory failure two months after discontinuing pembrolizumab due to repeated respiratory complaints. Imaging showed bilateral infiltrates and ground-glass opacities; infectious workup was negative. He was treated with corticosteroids for combined pembrolizumab-induced pneumonitis and diuretics for concurrent chronic heart failure exacerbation. Symptoms improved with supportive care and immunotherapy suspension. This case highlights the diagnostic challenge of differentiating irAE pneumonitis from infection or congestive heart failure (CHF) and underscores the need for early recognition in high-risk patients.

## Introduction

Immune checkpoint inhibitors, such as pembrolizumab, have revolutionized cancer therapy by improving survival across a range of malignancies. However, their increasing use has brought a parallel rise in immune-related adverse events (irAEs), which can involve multiple organ systems, including the lungs and heart [1,2]. In clinical trials, pembrolizumab-induced pneumonitis occurs in approximately 3-5% of patients, with severe cases reported in 1-3% [1,2]. Among these, pneumonitis is a potentially life-threatening complication that remains challenging to diagnose, particularly in patients with overlapping cardiopulmonary comorbidities. It may present with nonspecific respiratory symptoms and imaging findings that mimic infections or heart failure, often leading to delays in appropriate treatment [1,2]. This report presents a case of pembrolizumab-induced pneumonitis contributing to congestive heart failure (CHF) exacerbation in a patient with known cardiovascular disease. The absence of infectious findings and clinical improvement with immunosuppression and guideline-directed CHF therapy further implicates pembrolizumab as the likely etiology. As immune checkpoint inhibitors become increasingly integrated into cancer care, heightened awareness of their cardiopulmonary complications is essential for timely recognition and management.

## Case presentation

A 66-year-old male with a history of left-sided renal cell carcinoma on pembrolizumab (9 months, off for 2 months), hypertension, hyperlipidemia, coronary artery disease (s/p coronary artery bypass graft), heart failure-reduced ejection fraction (left ventricular ejection fraction 35%), atrial fibrillation with pacemaker, type II diabetes, and tophaceous gout presented with sudden-onset dyspnea. He denied fever, recent travel, or sick contacts but had been treated for influenza pneumonia two weeks prior.

On admission, blood pressure was 115/70 mmHg, heart rate was 83 bpm, respiratory rate (RR) was 18 breaths/min, and temperature was 97.6°F. He soon developed tachycardia (130 bpm), tachypnea (28 breaths/min), and hypoxia (peripheral oxygen saturation (SpO₂) 70%), requiring bilevel positive airway pressure (BiPAP) (fraction of inspired oxygen (FiO₂) 100%, 14/8, RR 16), which rapidly improved oxygenation (SpO₂ 100%). After two hours, the heart rate and respiratory rate improved to 89 and 19, respectively. The exam revealed diffuse wheezing, tachycardia, no edema, and multiple tophi on the extremities.

**Figure 1:**
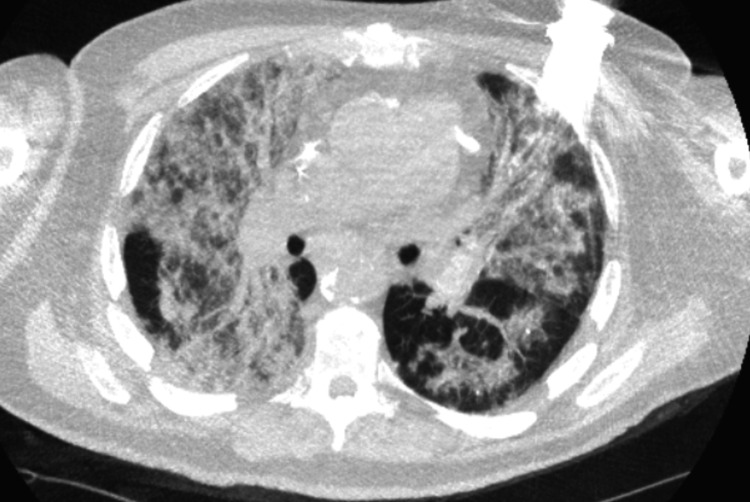
Chest CT demonstrating diffuse bilateral ground-glass opacities and interstitial changes consistent with immune checkpoint inhibitor–associated pneumonitis.

Labs showed leukocytosis (22 × 10⁹/L), lactate dehydrogenase (LDH) 490, B-type natriuretic peptide (BNP) 11,100, negative COVID-19 and respiratory viral panels (Table 1). Chest X-ray demonstrated bilateral infiltrates concerning for multifocal pneumonia versus pulmonary edema. CT chest revealed patchy ground-glass opacities and interstitial fibrosis, raising suspicion for pneumonitis versus chronic heart failure exacerbation (Figure 1).

**Table 1:** Pertinent laboratory findings on admission

Laboratory Test	Result	Reference Range
White Blood Cell Count	22 × 10⁹/L	4.0 – 10.0 × 10⁹/L
Lactate Dehydrogenase (LDH)	490 U/L	135 – 225 U/L
B-type Natriuretic Peptide (BNP)	11,100 pg/mL	<100 pg/mL

The patient was started on cefepime, doxycycline, and prednisone (20 mg daily) for suspected pembrolizumab-induced pneumonitis. Given concerns for pulmonary edema, IV furosemide was administered. A full septic workup, including blood cultures and urine testing for *Legionella* and *Streptococcus pneumoniae*, was negative. Left heart catheterization revealed no significant coronary artery stenoses. Due to high suspicion for pembrolizumab-induced pneumonitis, immunotherapy was temporarily put on hold. The patient’s symptoms improved over five days, allowing for prednisone tapering.

## Discussion

Pembrolizumab-related pneumonitis is a rare but serious irAE, occurring in 3-5% of patients receiving immune checkpoint inhibitors [1,3]. This inflammatory process can mimic infections or exacerbate comorbid conditions such as CHF [1,3]. The absence of an infectious source, confirmed by negative COVID-19 and microbiological testing, strongly implicates pembrolizumab-induced pneumonitis as a primary factor.

CHF exacerbation in this setting may result from inflammatory cytokine release, increased metabolic demand, and pulmonary fluid shifts [4,5]. Additionally, chronic interstitial fibrosis on CT suggests cumulative pulmonary damage from prior treatments or occupational exposures. Patients with pre-existing cardiac conditions are at increased risk for cardiopulmonary irAEs, highlighting the need for close monitoring and multidisciplinary management [2,6].

## Conclusions

This case illustrates the importance of recognizing immune-related pneumonitis in patients with underlying cardiac disease. The absence of infectious findings reinforces the role of immune checkpoint inhibitors in precipitating cardiopulmonary decompensation. Temporary immunotherapy suspension, corticosteroid therapy, and aggressive CHF management were effective in stabilizing this patient. This case highlights how immune-related pneumonitis can mimic or exacerbate heart failure in a patient without prior pulmonary toxicity - an underrecognized intersection with important implications for both oncology and cardiology teams. A limitation of this case is the lack of histopathologic confirmation, as the diagnosis was based on clinical, radiographic, and treatment response criteria. Also, follow-up imaging prior to discharge would have proven to be beneficial. Ultimately, clinicians should consider immune-related adverse events in patients on or recently off immunotherapy, as early differentiation from infectious or cardiac causes is essential for timely and appropriate treatment.
